# From Our Foreign Correspondent

**Published:** 1984-07

**Authors:** 


### From our Foreign Correspondent

### Ode to a Black Swan

Naked they came to that smooth-swarded bower,
And at their feet the crocus brake like fire,
Violet, amaracus, and asphodel,
Lotos and lilies.

Tennyson, Oenone

The clarity of the skies and the nude volleyball were among my first impressions of Perth. I had stopped for a few days at Singapore on the way out, with a brief excursion across the Straits of Johor and up to Malacca. Although within a stone's throw of the Equator, one was seldom aware of the sun; the steamy atmosphere and Raffles gin slings might explain this fact. Certainly it is sobering to arrive in a new country at 4 am, but when the sun came up in Australia I felt as if a curtain had lifted. The light made me blink and remember that I had forgotten my sunglasses. That first night, when searching the heavens for the Southern Cross, I thought I had never seen so many stars. The Milky Way was clearer than on a frosty night in January back home, and outdoor astronomy was a good deal more congenial.

Perhaps I should explain the volleyball. Childhood in Brighton has imbued me with a constant yearning to get close to the sea, a yearning which proximity to the Bristol Channel can somehow never satisfy. On my first day in Western Australia my kindly host took me for an afternoon constitutional on Swanbourne Beach. We rolled our trousers up above the knees and paddled along for a mile or two through the shallow surf of the Indian Ocean. After a while I became aware nobody else even had trousers on, for this it appeared was the local nudist beach. It was also an army firing range, which explained the red flags and agitated soldiery we spied on our return. A mile to the South lies Cottesloe Beach, where Prince Charles was kissed by a model and where the bus from my hall of residence reached the sea.

My reason for going to Perth was to take up a Raine Visiting Professorship at the University of Western Australia. Ma Raine was a barmaid from Liverpool who stepped off the boat at Fremantle and stayed on to acquire a string of hotels in Perth. The advent of the Second World War and the U.S. Navy made this a lucrative enterprise. The enormous Raine legacy allows the Medical Faculty to invite a steady succession of visitors from all over the world. This happy windfall is very necessary, for Perth is extraordinarily remote: Antarctica is about as close as Sydney, and neither intrude. Here lies the lotus charm of the Swan River and its environs. Perhaps two months was a long enough sojourn, or I might have had difficulty in tearing myself away. Inevitably the geographical situation breeds a sturdy insularity. Most doctors in W.A. are local men who train in Perth and practise there throughout their professional career. The dangers are obvious but so are the attractions. There are of course several British emigres, and I met two men I had not seen since medical school.

I arrived in September, which is Springtime on the Swan. The wild flowers were spectacular. They grow in profusion in King's Park, a large open space which lies between the University in Nedlands and the city centre. Particularly memorable was the kangaroo paw, a sort of dwarf triffid, which is the state flower. Later, on a visit to Margaret River in the extreme South West, I saw actual kangaroo paw prints in the sand as well as their biped owners which come out at night. The wild flowers there included orchids and innumerable arum lilies. There are lots of different banksia trees and a strange, primaeval, tree-like plant called blackboy. The blackboy grows exceedingly slowly; its woody and fibrous trunk is used to make distinctive ornaments. When the Commonwealth Games came to Perth, the government tried valiantly but unsuccessfully to persuade the populace to call it tree-grass.

I had left England halfway through the America's Cup series, with the U.S.A. leading 3—1. With some trepidation I asked for news from Newport at Perth Airport. It was 3-all, with the deciding race the following night. The yachts were locked in combat off Rhode Island at 3 am local time, so I went to bed. When I awoke next day there was a deathly hush, and I feared the worst. It turned out the race had been cancelled. When I awoke after the re-run, it was obvious at once which side had won. Prised from its Yankee plinth, the Cup was brought with much joy to the Royal Perth Yacht Club, just down the road. The blustery winds between Fremantle and Rottnest Island will ensure that the challengers in 1987 have a difficult job to succeed.

What did my job entail, you may wonder. I was based at the main teaching hospital, the Sir Charles Gairdner. This is a new building and magnificently equipped. It is rivalled in luxury only by a large private hospital, the St John of God in Subiaco. I spent one week at Fremantle Hospital and two at the Royal Perth in the city centre. I took a number of student teaching rounds, attended research and postgraduate sessions and gave about a dozen lectures during my 8-week stay (500 slides had weighed down my briefcase). I went to theatre a few times and operated once. I acted as external examiner in the Finals, which are taken in November. I was flown by an anaesthetist to Geraldton and took the Prospector train to Kalgoorlie, visiting the regional hospital in each town. I also had a week at a meeting in Melbourne and Adelaide. Everywhere I received amazing hospitality. If everyone I invited back to Bristol takes me up on the offer, we shall need to set up a small hotel. Space does not allow me to compare Australian and British medicine in an adequate fashion, so I shall not embark on the task.

Just before I left Perth my wife joined me, and we continued around the world. We stopped in Sydney, Brisbane, Auckland and Tahiti and finished our Christmas shopping in Los Angeles. Although it was nice to be back, I sometimes long for the weather of Western Australia and the sandy beaches of the Indian Ocean.

Robin Williamson

